# 3-(3-Chloro­prop­yl)-7,8-dimeth­oxy-2,3,4,5-tetra­hydro-1*H*-3-benzazepin-2-one at 125 K

**DOI:** 10.1107/S1600536808008787

**Published:** 2008-04-04

**Authors:** Xiang-Wei Cheng

**Affiliations:** a Zhejiang Police College Experience Center, Zhejiang Police College, Hangzhou 310053, People’s Republic of China

## Abstract

In the title compound, C_15_H_20_ClNO_3_, the seven-membered ring adopts a distorted boat–sofa conformation; the methyl­ene C atoms of this ring are coplanar with the benzene ring. Both meth­oxy groups are almost coplanar with the attached benzene ring [C—C—O—C = 6.5 (2) and −13.5 (3)°]. An intra­molecular C—H⋯O hydrogen bond is observed in the mol­ecular structure. In the crystal structure, a C—H⋯π inter­action involving the benzene ring is observed.

## Related literature

For details of the synthesis, see: Reiffen *et al.* (1981 ▶). For general background, see: Ishihara *et al.* (1994 ▶). For a related structure, see: Reiffen *et al.* (1990 ▶).
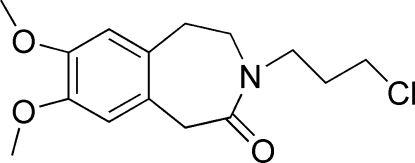


## Experimental

### 

#### Crystal data

C_15_H_20_ClNO_3_
*M* *_r_* = 297.77
Triclinic, 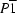

*a* = 8.134 (3) Å
*b* = 8.498 (3) Å
*c* = 11.701 (4) Å
α = 92.880 (12)°
β = 105.981 (12)°
γ = 106.440 (12)°
*V* = 738.5 (5) Å^3^
*Z* = 2
Mo *K*α radiation
μ = 0.27 mm^−1^
*T* = 125 K
0.29 × 0.28 × 0.22 mm

#### Data collection

Bruker SMART CCD area-detector diffractometer
Absorption correction: multi-scan (*SADABS*; Bruker, 2002 ▶) *T*_min_ = 0.927, *T*_max_ = 0.944
6809 measured reflections
2542 independent reflections
2137 reflections with *I* > 2σ(*I*)
*R*_int_ = 0.020

#### Refinement

*R*[*F* ^2^ > 2σ(*F* ^2^)] = 0.039
*wR*(*F* ^2^) = 0.112
*S* = 1.06
2542 reflections
183 parameters
H-atom parameters constrained
Δρ_max_ = 0.19 e Å^−3^
Δρ_min_ = −0.23 e Å^−3^

### 

Data collection: *SMART* (Bruker, 2002 ▶); cell refinement: *SAINT* (Bruker, 2002 ▶); data reduction: *SAINT*; program(s) used to solve structure: *SHELXS97* (Sheldrick, 2008 ▶); program(s) used to refine structure: *SHELXL97* (Sheldrick, 2008 ▶); molecular graphics: *SHELXTL* (Sheldrick, 2008 ▶); software used to prepare material for publication: *SHELXTL*.

## Supplementary Material

Crystal structure: contains datablocks global, I. DOI: 10.1107/S1600536808008787/ci2575sup1.cif

Structure factors: contains datablocks I. DOI: 10.1107/S1600536808008787/ci2575Isup2.hkl

Additional supplementary materials:  crystallographic information; 3D view; checkCIF report

## Figures and Tables

**Table 1:** Hydrogen-bond geometry (Å, °)

*D*—H⋯*A*	*D*—H	H⋯*A*	*D*⋯*A*	*D*—H⋯*A*
C14—H14*A*⋯O3	0.97	2.36	2.768 (2)	104
C1—H1*A*⋯*Cg*1	0.96	2.84	3.705 (3)	150

*Cg*1 is the centroid of the benzene ring.

**Fig. 1.**
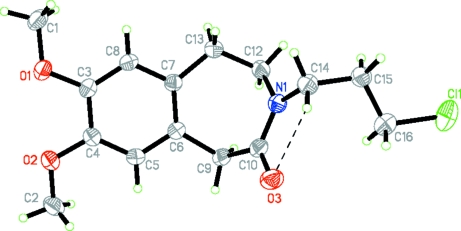
Molecular structure of the title compound, showing 30% probability displacement ellipsoids and the atomic numbering.

**Fig. 2.**
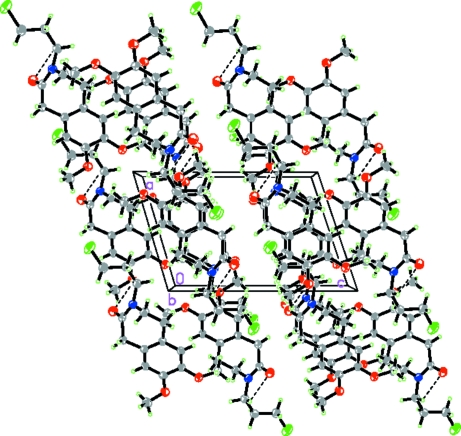
Crystal packing of the title compound, viewed approximately down the b axis. Dashed lines indicate intramolecular hydrogen bonds.

## supplementary crystallographic information

### Comment

Benzazepine derivatives have been of considerable medicinal interest, partly because the skeleton is a component of amaryllydaceae alkaloids such as galanthamine as well as of ribasine alkaloids represented by ribasine (Ishihara *et al.*, 1994). Many benzazepine derivatives have been reported to possess interesting biological activities. The title compound is an important intermediate of ivabradine, which was listed in market in 2006 as the representative of a novel pharmacological class termed specific bradycardic agents. Here the crystal structure of the title compound is reported.

In the title molecule (Fig.1), the seven-membered ring adopts a distorted boat-sofa conformation with a pseudo mirror plane through C7 and the centre of the N1—C10 bond. Atoms C9, C12 and C13 of the seven-membered ring are coplanar with the benzene ring. The dihedral angle between the C3-C9/C12/C13 and C9/C10/N1/C12 planes is 60.61 (8)°. The chloropropyl substituent group is in a (-)-synclinal conformation, as evidenced by the torsion angle N1—C14—C15—C16 of -68.9 (2)°, similar to that in a related structure (-62.63 (2)°, Reiffen *et al.*, 1990). The methoxy groups are almost coplanar with the benzene ring [C8—C3—O1—C1 = 6.5 (2)° and C5—C4—O2—C2 = -13.5 (3)°].

An intramolecular C—H···O hydrogen bond is observed in the molecular structure (Fig.1). In the crystal structure, a C—H···π interaction involving the benzene ring is observed (Table 1).

### Experimental

The title compound was prepared according to the literature method (Reiffen *et al.*, 1981). Crystals suitable for X-ray analysis were obtained by slow evaporation of an isopropanol solution at 295 K.

### Refinement

H atoms were positioned geometrically (C-H = 0.93-0.97 Å) and refined using a riding model, with *U*_iso_(H) = 1.2–1.5*U*_eq_(C).

### Crystal data

**Table:**

C_15_H_20_ClNO_3_	*Z* = 2
*M**_r_* = 297.77	*F*_000_ = 316
Triclinic, *P*1	*D*_x_ = 1.339 Mg m^−^^3^
Hall symbol: -P 1	Mo *K*α radiation λ = 0.71073 Å
*a* = 8.134 (3) Å	Cell parameters from 2542 reflections
*b* = 8.498 (3) Å	θ = 1.8–25.0º
*c* = 11.701 (4) Å	µ = 0.27 mm^−^^1^
α = 92.880 (12)º	*T* = 125 K
β = 105.981 (12)º	Block, colourless
γ = 106.440 (12)º	0.29 × 0.28 × 0.22 mm
*V* = 738.5 (5) Å^3^	

### Data collection

**Table:**

Bruker SMART CCD area-detector diffractometer	2542 independent reflections
Radiation source: fine-focus sealed tube	2137 reflections with *I* > 2σ(*I*)
Monochromator: graphite	*R*_int_ = 0.020
*T* = 123(2) K	θ_max_ = 25.0º
φ and ω scans	θ_min_ = 1.8º
Absorption correction: multi-scan(SADABS; Bruker, 2002)	*h* = −9→9
*T*_min_ = 0.927, *T*_max_ = 0.944	*k* = −9→9
6809 measured reflections	*l* = −13→11

### Refinement

**Table:**

Refinement on *F*^2^	Secondary atom site location: difference Fourier map
Least-squares matrix: full	Hydrogen site location: inferred from neighbouring sites
*R*[*F*^2^ > 2σ(*F*^2^)] = 0.039	H-atom parameters constrained
*wR*(*F*^2^) = 0.112	*w* = 1/[σ^2^(*F*_o_^2^) + (0.0588*P*)^2^ + 0.1232*P*] where *P* = (*F*_o_^2^ + 2*F*_c_^2^)/3
*S* = 1.06	(Δ/σ)_max_ = 0.001
2542 reflections	Δρ_max_ = 0.19 e Å^−^^3^
183 parameters	Δρ_min_ = −0.23 e Å^−^^3^
Primary atom site location: structure-invariant direct methods	Extinction correction: none

### Special details

**Table:**

Geometry. All e.s.d.'s (except the e.s.d. in the dihedral angle between two l.s. planes) are estimated using the full covariance matrix. The cell e.s.d.'s are taken into account individually in the estimation of e.s.d.'s in distances, angles and torsion angles; correlations between e.s.d.'s in cell parameters are only used when they are defined by crystal symmetry. An approximate (isotropic) treatment of cell e.s.d.'s is used for estimating e.s.d.'s involving l.s. planes.
Refinement. Refinement of *F*^2^ against ALL reflections. The weighted *R*-factor *wR* and goodness of fit *S* are based on *F*^2^, conventional *R*-factors *R* are based on *F*, with *F* set to zero for negative *F*^2^. The threshold expression of *F*^2^ > σ(*F*^2^) is used only for calculating *R*-factors(gt) *etc*. and is not relevant to the choice of reflections for refinement. *R*-factors based on *F*^2^ are statistically about twice as large as those based on *F*, and *R*- factors based on ALL data will be even larger.

### Fractional atomic coordinates and isotropic or equivalent isotropic displacement parameters (Å^2^)

**Table:**

	*x*	*y*	*z*	*U*_iso_*/*U*_eq_	
C1	0.2530 (3)	0.0704 (3)	1.09762 (17)	0.0633 (5)	
H1A	0.3722	0.0674	1.1371	0.095*	
H1B	0.1732	0.0182	1.1413	0.095*	
H1C	0.2544	0.1834	1.0945	0.095*	
C2	−0.0455 (3)	−0.2072 (3)	0.63151 (18)	0.0689 (6)	
H2A	−0.0677	−0.1141	0.5942	0.103*	
H2B	−0.1580	−0.2894	0.6243	0.103*	
H2C	0.0232	−0.2539	0.5927	0.103*	
C3	0.2849 (2)	0.0480 (2)	0.90096 (14)	0.0445 (4)	
C4	0.2103 (2)	−0.02661 (19)	0.78048 (15)	0.0445 (4)	
C5	0.2973 (2)	0.0306 (2)	0.69805 (14)	0.0450 (4)	
H5	0.2487	−0.0210	0.6189	0.054*	
C6	0.4565 (2)	0.1640 (2)	0.72932 (14)	0.0430 (4)	
C7	0.5290 (2)	0.2400 (2)	0.84825 (14)	0.0439 (4)	
C8	0.4413 (2)	0.1789 (2)	0.93214 (14)	0.0451 (4)	
H8	0.4907	0.2287	1.0117	0.054*	
C9	0.5404 (2)	0.2131 (2)	0.62888 (15)	0.0501 (4)	
H9A	0.4721	0.1345	0.5568	0.060*	
H9B	0.5321	0.3214	0.6114	0.060*	
C10	0.7353 (2)	0.2180 (2)	0.66095 (15)	0.0493 (4)	
N1	0.85388 (18)	0.33947 (17)	0.74910 (12)	0.0489 (4)	
C12	0.7942 (2)	0.4582 (2)	0.80910 (17)	0.0539 (5)	
H12A	0.7125	0.4977	0.7491	0.065*	
H12B	0.8975	0.5526	0.8506	0.065*	
C13	0.7006 (2)	0.3845 (2)	0.89834 (16)	0.0556 (5)	
H13A	0.6725	0.4718	0.9383	0.067*	
H13B	0.7848	0.3482	0.9589	0.067*	
C14	1.0354 (2)	0.3319 (2)	0.80602 (16)	0.0544 (4)	
H14A	1.0420	0.2247	0.7789	0.065*	
H14B	1.0568	0.3403	0.8922	0.065*	
C15	1.1839 (2)	0.4668 (2)	0.77991 (16)	0.0556 (5)	
H15A	1.1704	0.5743	0.7980	0.067*	
H15B	1.3001	0.4661	0.8307	0.067*	
C16	1.1757 (3)	0.4392 (3)	0.65120 (18)	0.0636 (5)	
H16A	1.0595	0.4403	0.6005	0.076*	
H16B	1.1884	0.3314	0.6332	0.076*	
O1	0.19242 (16)	−0.01575 (16)	0.97846 (11)	0.0577 (3)	
O2	0.05252 (16)	−0.15427 (16)	0.75527 (11)	0.0600 (4)	
O3	0.78269 (18)	0.11586 (17)	0.61105 (12)	0.0660 (4)	
Cl1	1.35151 (9)	0.59760 (9)	0.61897 (6)	0.0942 (3)	

### Atomic displacement parameters (Å^2^)

**Table:**

	*U*^11^	*U*^22^	*U*^33^	*U*^12^	*U*^13^	*U*^23^
C1	0.0704 (12)	0.0768 (13)	0.0502 (11)	0.0225 (10)	0.0293 (9)	0.0132 (9)
C2	0.0645 (12)	0.0601 (11)	0.0607 (12)	0.0006 (9)	0.0060 (9)	−0.0011 (9)
C3	0.0455 (9)	0.0489 (9)	0.0460 (9)	0.0206 (7)	0.0177 (7)	0.0124 (7)
C4	0.0421 (8)	0.0410 (8)	0.0499 (9)	0.0140 (7)	0.0120 (7)	0.0066 (7)
C5	0.0470 (9)	0.0471 (9)	0.0397 (9)	0.0171 (7)	0.0095 (7)	0.0026 (7)
C6	0.0453 (9)	0.0453 (9)	0.0416 (9)	0.0180 (7)	0.0137 (7)	0.0076 (7)
C7	0.0451 (9)	0.0439 (8)	0.0442 (9)	0.0160 (7)	0.0141 (7)	0.0041 (7)
C8	0.0472 (9)	0.0512 (9)	0.0378 (8)	0.0180 (7)	0.0121 (7)	0.0028 (7)
C9	0.0516 (10)	0.0584 (10)	0.0392 (9)	0.0150 (8)	0.0142 (7)	0.0059 (7)
C10	0.0556 (10)	0.0540 (10)	0.0420 (9)	0.0171 (8)	0.0199 (8)	0.0094 (8)
N1	0.0456 (8)	0.0517 (8)	0.0505 (8)	0.0153 (6)	0.0166 (6)	0.0040 (6)
C12	0.0520 (10)	0.0473 (9)	0.0595 (11)	0.0093 (8)	0.0198 (8)	0.0011 (8)
C13	0.0565 (10)	0.0529 (10)	0.0523 (10)	0.0074 (8)	0.0200 (8)	−0.0039 (8)
C14	0.0516 (10)	0.0603 (11)	0.0518 (10)	0.0201 (8)	0.0133 (8)	0.0098 (8)
C15	0.0487 (9)	0.0630 (11)	0.0538 (11)	0.0186 (8)	0.0126 (8)	0.0048 (8)
C16	0.0696 (12)	0.0699 (12)	0.0594 (12)	0.0288 (10)	0.0245 (10)	0.0111 (9)
O1	0.0544 (7)	0.0688 (8)	0.0494 (7)	0.0114 (6)	0.0225 (6)	0.0116 (6)
O2	0.0532 (7)	0.0581 (7)	0.0558 (7)	0.0010 (6)	0.0136 (6)	0.0044 (6)
O3	0.0664 (8)	0.0721 (9)	0.0619 (8)	0.0255 (7)	0.0217 (7)	−0.0058 (7)
Cl1	0.1040 (5)	0.0999 (5)	0.1101 (5)	0.0398 (4)	0.0683 (4)	0.0438 (4)

### Geometric parameters (Å, °)

**Table:**

C1—O1	1.428 (2)	C9—H9A	0.97
C1—H1A	0.96	C9—H9B	0.97
C1—H1B	0.96	C10—O3	1.227 (2)
C1—H1C	0.96	C10—N1	1.357 (2)
C2—O2	1.425 (2)	N1—C12	1.463 (2)
C2—H2A	0.96	N1—C14	1.465 (2)
C2—H2B	0.96	C12—C13	1.515 (2)
C2—H2C	0.96	C12—H12A	0.97
C3—O1	1.3697 (19)	C12—H12B	0.97
C3—C8	1.375 (2)	C13—H13A	0.97
C3—C4	1.407 (2)	C13—H13B	0.97
C4—O2	1.372 (2)	C14—C15	1.524 (3)
C4—C5	1.377 (2)	C14—H14A	0.97
C5—C6	1.401 (2)	C14—H14B	0.97
C5—H5	0.93	C15—C16	1.492 (3)
C6—C7	1.394 (2)	C15—H15A	0.97
C6—C9	1.533 (2)	C15—H15B	0.97
C7—C8	1.403 (2)	C16—Cl1	1.805 (2)
C7—C13	1.519 (2)	C16—H16A	0.97
C8—H8	0.93	C16—H16B	0.97
C9—C10	1.513 (2)		
			
O1—C1—H1A	109.5	O3—C10—C9	121.63 (16)
O1—C1—H1B	109.5	N1—C10—C9	116.40 (15)
H1A—C1—H1B	109.5	C10—N1—C12	121.13 (14)
O1—C1—H1C	109.5	C10—N1—C14	120.32 (15)
H1A—C1—H1C	109.5	C12—N1—C14	117.01 (14)
H1B—C1—H1C	109.5	N1—C12—C13	112.78 (15)
O2—C2—H2A	109.5	N1—C12—H12A	109.0
O2—C2—H2B	109.5	C13—C12—H12A	109.0
H2A—C2—H2B	109.5	N1—C12—H12B	109.0
O2—C2—H2C	109.5	C13—C12—H12B	109.0
H2A—C2—H2C	109.5	H12A—C12—H12B	107.8
H2B—C2—H2C	109.5	C12—C13—C7	116.64 (15)
O1—C3—C8	124.96 (15)	C12—C13—H13A	108.1
O1—C3—C4	116.39 (14)	C7—C13—H13A	108.1
C8—C3—C4	118.64 (15)	C12—C13—H13B	108.1
O2—C4—C5	125.10 (15)	C7—C13—H13B	108.1
O2—C4—C3	115.54 (15)	H13A—C13—H13B	107.3
C5—C4—C3	119.36 (14)	N1—C14—C15	114.14 (15)
C4—C5—C6	122.21 (15)	N1—C14—H14A	108.7
C4—C5—H5	118.9	C15—C14—H14A	108.7
C6—C5—H5	118.9	N1—C14—H14B	108.7
C7—C6—C5	118.52 (15)	C15—C14—H14B	108.7
C7—C6—C9	124.80 (14)	H14A—C14—H14B	107.6
C5—C6—C9	116.68 (14)	C16—C15—C14	110.34 (15)
C6—C7—C8	118.89 (15)	C16—C15—H15A	109.6
C6—C7—C13	125.75 (15)	C14—C15—H15A	109.6
C8—C7—C13	115.34 (14)	C16—C15—H15B	109.6
C3—C8—C7	122.35 (15)	C14—C15—H15B	109.6
C3—C8—H8	118.8	H15A—C15—H15B	108.1
C7—C8—H8	118.8	C15—C16—Cl1	110.88 (14)
C10—C9—C6	112.81 (14)	C15—C16—H16A	109.5
C10—C9—H9A	109.0	Cl1—C16—H16A	109.5
C6—C9—H9A	109.0	C15—C16—H16B	109.5
C10—C9—H9B	109.0	Cl1—C16—H16B	109.5
C6—C9—H9B	109.0	H16A—C16—H16B	108.1
H9A—C9—H9B	107.8	C3—O1—C1	117.37 (14)
O3—C10—N1	121.96 (16)	C4—O2—C2	116.36 (14)
			
O1—C3—C4—O2	0.3 (2)	C6—C9—C10—N1	−68.2 (2)
C8—C3—C4—O2	−178.75 (15)	O3—C10—N1—C12	−178.83 (16)
O1—C3—C4—C5	−179.49 (14)	C9—C10—N1—C12	0.1 (2)
C8—C3—C4—C5	1.4 (2)	O3—C10—N1—C14	−13.4 (3)
O2—C4—C5—C6	178.70 (15)	C9—C10—N1—C14	165.52 (15)
C3—C4—C5—C6	−1.5 (3)	C10—N1—C12—C13	75.6 (2)
C4—C5—C6—C7	0.3 (3)	C14—N1—C12—C13	−90.34 (18)
C4—C5—C6—C9	179.18 (15)	N1—C12—C13—C7	−61.8 (2)
C5—C6—C7—C8	0.9 (2)	C6—C7—C13—C12	3.9 (3)
C9—C6—C7—C8	−177.88 (15)	C8—C7—C13—C12	−177.43 (15)
C5—C6—C7—C13	179.47 (16)	C10—N1—C14—C15	112.96 (18)
C9—C6—C7—C13	0.7 (3)	C12—N1—C14—C15	−81.02 (19)
O1—C3—C8—C7	−179.23 (15)	N1—C14—C15—C16	−68.9 (2)
C4—C3—C8—C7	−0.3 (2)	C14—C15—C16—Cl1	−179.73 (13)
C6—C7—C8—C3	−0.9 (3)	C8—C3—O1—C1	6.5 (2)
C13—C7—C8—C3	−179.66 (16)	C4—C3—O1—C1	−172.49 (15)
C7—C6—C9—C10	52.6 (2)	C5—C4—O2—C2	−13.5 (3)
C5—C6—C9—C10	−126.15 (16)	C3—C4—O2—C2	166.68 (16)
C6—C9—C10—O3	110.71 (19)		

### Hydrogen-bond geometry (Å, °)

**Table:**

*D*—H···*A*	*D*—H	H···*A*	*D*···*A*	*D*—H···*A*
C14—H14A···O3	0.97	2.36	2.768 (2)	104
C1—H1A···Cg1	0.96	2.84	3.705 (3)	150
